# Cyclic Load Test and Finite Element Analysis of NOVEL Buckling-Restrained Brace

**DOI:** 10.3390/ma13225103

**Published:** 2020-11-12

**Authors:** Robel Wondimu Alemayehu, Youngsik Kim, Jaehoon Bae, Young K. Ju

**Affiliations:** 1 School of Civil, Environmental and Architectural Engineering, Korea University, Anamdong, Seongbukgu, Seoul 136713, Korea; robel@korea.ac.kr (R.W.A.); yskim@techsq.co.kr (Y.K.); skycitybjh80@gmail.com (J.B.); 2 Technical Research Center, TechSquare Co., Ltd., 25 Banpo-daero, Seocho-gu, Seoul 06710, Korea

**Keywords:** buckling-restrained brace, subassembly test, component test, finite element analysis, BRB global buckling, H-section core flange buckling

## Abstract

Compared to concrete or mortar-filled Buckling-Restrained Braces (BRBs), all-steel BRBs provide weight and fabrication time reductions. In particular, all-steel buckling braces with H-section cores are gaining attention in cases where large axial strength is required. In this paper, an all-steel BRB, called NOVEL (Noise, CO_2_ emission, Vibration, Energy dissipation and Labor), is presented. It comprises an H-section core encased in a square casing, and its behavior was studied through full-scale subassembly and brace tests, followed by a finite element parametric study. Two failure modes were observed: global buckling and flange buckling of the H-section core, which occurred in test specimens with *P_cr_/P_y_* ratios of 1.68 and 4.91, respectively. Global buckling occurred when the maximum moment in the casing reached its yielding moment, although the test specimens had sufficient stiffness to prevent global buckling. Failure by core flange buckling occurred at a core strain of 1.2%. The finite element parametric study indicated that adjusting the width-to-thickness ratio of the core flange is more feasible than stiffening the flange or adjusting the unconstrained-length end stiffeners. The value of 5.06 was the minimum flange slenderness ratio that provided a stable hysteresis to the end of the loading protocol of the American Institute of Steel Construction standard.

## 1. Introduction

Buckling-Restrained Braces (BRBs) have been widely implemented in framed structures to reduce damage during severe earthquakes [1,2]. Unlike conventional braces that buckle under compression, the core of BRBs yields both in tension and compression under the restraining effect of the casing. The casing should provide sufficient restraint against global buckling to ensure stable hysteresis and energy dissipation, and local buckling of the core and the casing should be avoided. 

Several researchers have proposed all-steel BRBs [3,4,5,6,7,8,9] over conventional concrete-filled BRBs for various advantages, such as lighter weight, shorter fabrication time, and, in some cases, disassembly for maintenance and inspection [3]. Some of the common cross-sectional shapes of all-steel BRBs include H-section core encased in a square or circular casing, cruciform core encased in a square casing, circular tube core encased in a circular tube casing, and flat rectangular core plate sandwiched between bolted casing plates [3,4]. When high BRB axial strength is required, H-section, cruciform, or circular hollow section cores are better alternatives than thick flat plate cores. In cruciform core sections, the presence of welding deformation, large initial imperfection, and residual stress reduce the fatigue life and assembly accuracy of BRBs [10]. Moreover, compared to cruciform and flat cores, H-section cores show higher stability [11]. Thus, researchers are focusing on the use of H-section BRB cores [10].

A typical BRB comprises an unconstrained core segment at the two ends of the casing. Previous test results [8,12] have shown the presence of large in-plane bending demand in the unconstrained segment of a BRB, being a potential failure zone.

**Figure 1:**
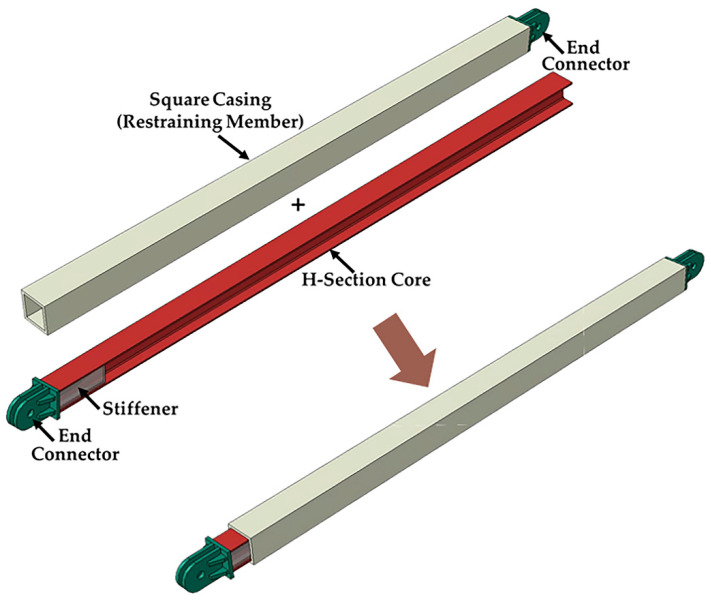
Noise, CO_2_ emission, Vibration, Energy dissipation and Labor (NOVEL) buckling-restrained brace.

In this paper, an all-steel BRB aiming to alleviate construction Noise, CO_2_ emission, Vibration, Energy dissipation and Labor (NOVEL), which comprises an H-section core and a square casing, is presented. The core, casing, and end connectors are arranged, as shown in Figure 1, to obtain an unconstrained length only on one side. The behavior of the brace is investigated through subassembly and brace tests followed by a finite element parametric study. 

## 2. Test Program

### 2.1. Test Specimens

**Figure 2:**
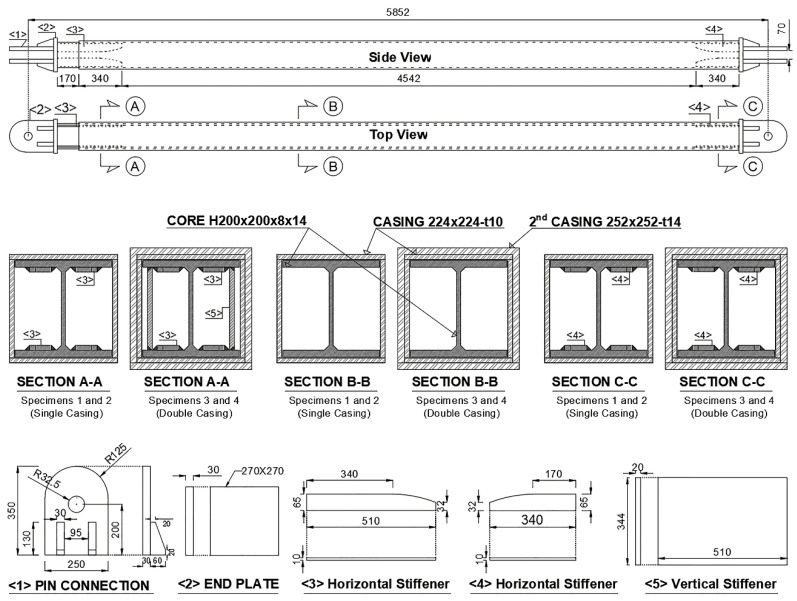
Details of test specimens.

The test program comprises cyclic load tests of four full-scale BRB specimens. Figure 2 shows the dimensions and details of the specimens, which had identical lengths and core sizes. Specimens 3 and 4 differ from Specimens 1 and 2 in the casing size and stiffener details only. Specimens 1 and 2 utilized a single casing and horizontal unconstrained-length stiffeners parallel to the flange of the core. Specimens 3 and 4 utilized double casing and vertical unconstrained-length stiffeners in addition to the horizontal stiffener, as shown in Figure 2. The dimensions of the casing of Specimens 1 and 2 and the inner casing of Specimens 3 and 4 were identical and are presented in Figure 2, which also summarizes the cross-sectional dimensions of the core, casing, and stiffeners, along with details of the end connectors.

The test specimens were fabricated using SS275 steel with a nominal yield strength of 275 MPa. Table 1 summarizes the average values of yield, ultimate strength, and elongation at rupture of the core and casing plates obtained from tensile coupon tests. Table 2 lists the stiffness and strength parameters computed using the coupon test results. Here, *P_y_* and *P_u_* represent the yield and tensile strengths of the core, respectively, obtained by multiplying the core cross-sectional area with the yield and tensile strengths from the coupon test, respectively. Further, *P_cr_* is Euler’s critical buckling load given by Equation (1), where *E*, *I*, and *L* are the elastic modulus, casing moment of inertia, and effective length of the buckling-restrained brace, respectively. The modulus of elasticity used for calculating the buckling load was 200 GPa. Watanabe et al. [13] recommended a *P_cr_/P_y_* ratio higher than 1.5 to prevent global buckling, which was applied, as shown in Table 2. The other casing strength parameter presented in Table 2 is the casing yielding moment *M_y_* calculated as the product of the casing elastic section modules and casing yield strength obtained from the coupon test. The maximum moment at the center of the casing and the brace axial force (*P*) are related by Equation (2) [14,15]. Further, the brace axial force to cause flexure yielding of the casing (*P_cy_*) is computed by setting *M_center_* to *M_y_*. Here, $\nu_{0}$ represents the sum of the initial imperfection amplitude and the gap between the core and casing, which were set as 12 mm (L/500) [16] and 2 mm, respectively. For convenient comparison, *P_cy_* is represented as a ratio of *P_y_* in Table 2.

(1) $$P_{cr}=\frac{\pi^{2}EI}{L^{2}}$$

(2) $$M_{center}=\frac{P\nu_{0}}{1-\frac{P}{P_{cr}}}$$

### 2.2. Test Setup

**Figure 3:**
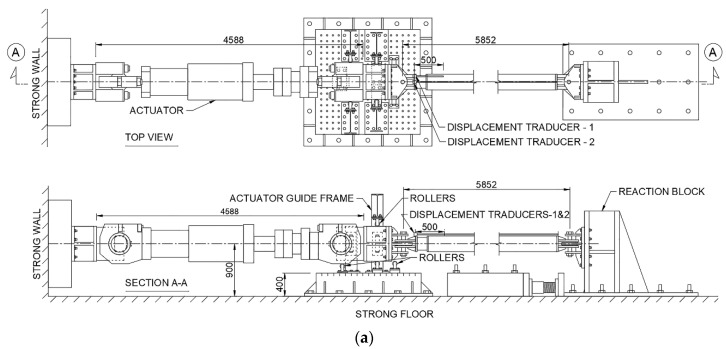

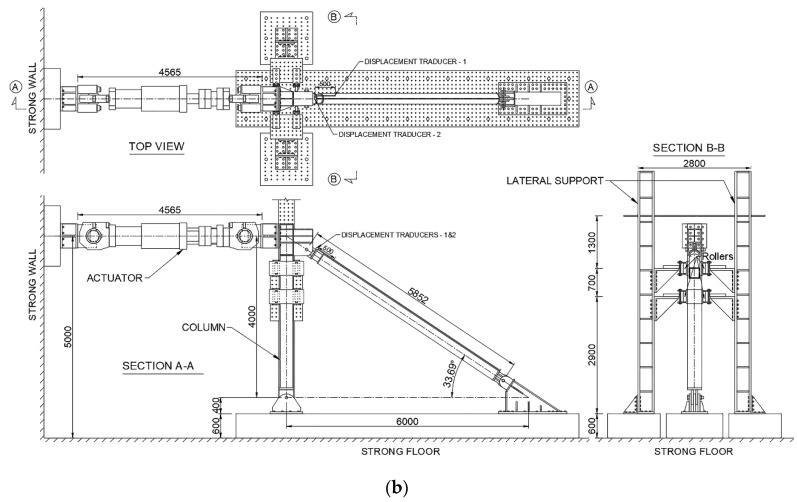
Test setup: (**a**) brace test and (**b**) subassembly test.

The test was conducted in the two setups shown in Figure 3a and b using a 3000 kN hydraulic actuator. Specimens 1–3 were tested in the brace test setup shown in Figure 3a, whereas Specimen 4 was tested in the subassembly test setup shown in Figure 3b. In the brace test setup, the BRB was positioned horizontally, and pin-connected to the actuator and a reaction block on the two ends. The out-of-plane and vertical movement of the actuator was prevented by a set of roller supports that fitted the width and depth of the actuator head, as shown in Figure 3a. In the subassembly test setup, the BRB specimen was positioned at a 33.69° inclination angle (4 m story height and 6 m span), and pin-connected to a column, as shown in Figure 3b. The opposite end of the BRB specimen and the column base were pin-supported to the laboratory strong floor. The out-of-plane movement of the assembly was restrained by a lateral support frame, which was connected to the column through a set of rollers that fitted the width of the column. Displacement transducers 1 and 2, shown in Figure 3a,b, were used to measure the relative displacement between the core and the casing, and the relative displacement between the brace ends, respectively.

### 2.3. Loading Protocol

**Figure 4:**
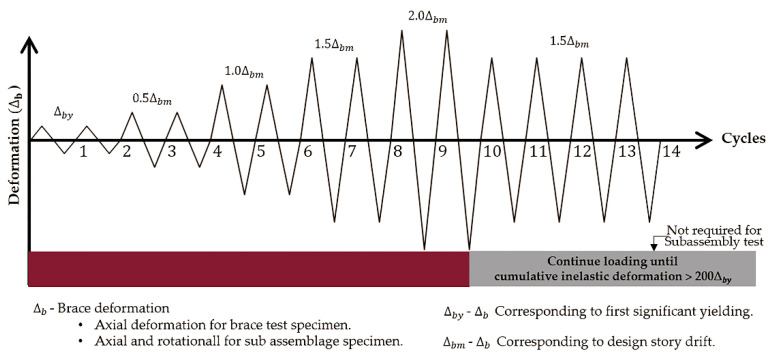
Loading protocol.

The quasi-static cyclic loading protocol specified in the American Institute of Steel Construction seismic provision [17] is shown in Figure 4. The loading protocol starts with two cycles of *Δ_by_*, followed by two cycles of 0.5*Δ_bm_*, 1.5*Δ_bm_*, and 2.0*Δ_bm_* each, where *Δ_by_* and *Δ_bm_* indicate the displacement corresponding to core yielding and design story drift, respectively. In this test, *Δ_bm_* was set to 1.0% of the story height [17], and *Δ_by_* was calculated as a 7.3 mm brace axial displacement based on the yielding length of the core and the yield strength obtained from the coupon tests. The modulus of elasticity was set to 200 GPa. Following the two 2.0*Δ_bm_* cycles, the loading protocol continues with fatigue loading at 1.5*Δ_bm_* loading amplitude until the cumulative inelastic deformation exceeds 200*Δ_by_*. Fatigue loading is not required for the subassembly test setup [17].

**Figure 5:**
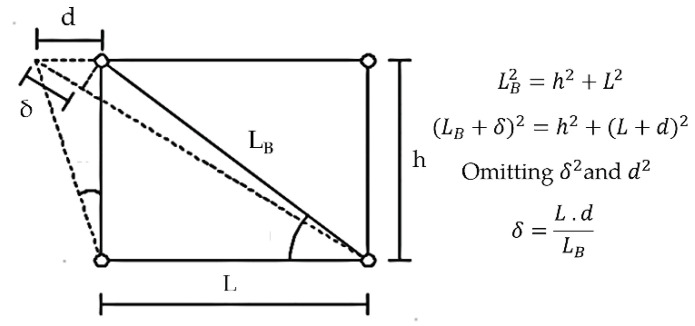
Story drift and brace axial displacement correlation.

Figure 5 and Equation (3) show the correlation between the brace axial deformation (*δ*) and the story drift (d). During all tests, *δ* was monitored using a displacement transducer. In the subassembly test setup with 4 m story height, d corresponding to 2.0*Δ_bm_* was 80 mm. The corresponding *δ* was 66.56 mm. The same value of *δ* was adopted for the brace test of Specimen 1, whereas it was reduced to 53.25 mm for Specimens 2 and 3, assuming a 3.2 m story height.

(3) $$\delta =\frac{L\cdot d}{L_{B}}$$

## 3. Test Results

**Figure 6:**
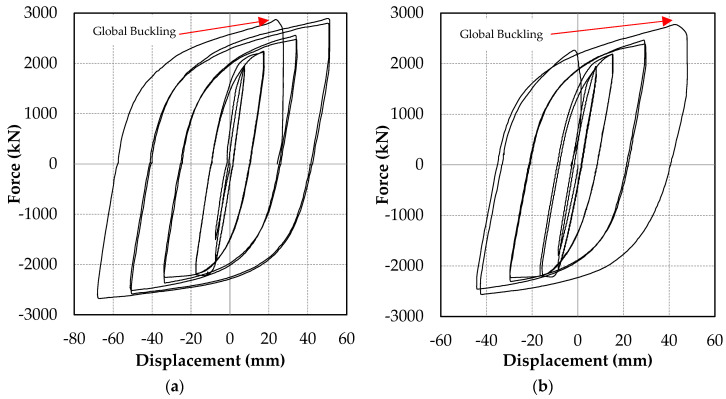

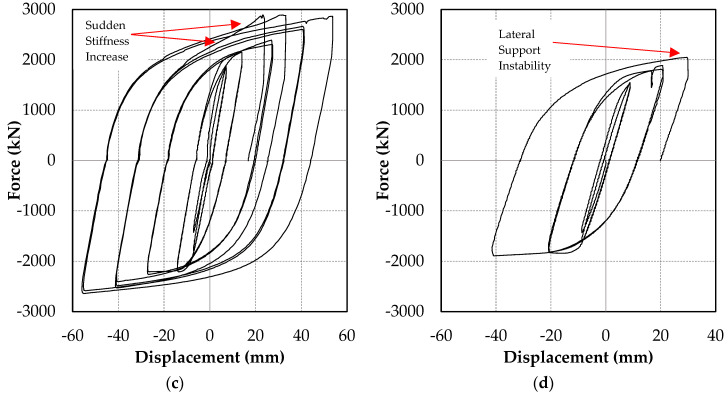
Hysteretic responses. Specimens (**a**) 1, (**b**) 2, (**c**) 3, and (**d**) 4.

**Figure 7:**
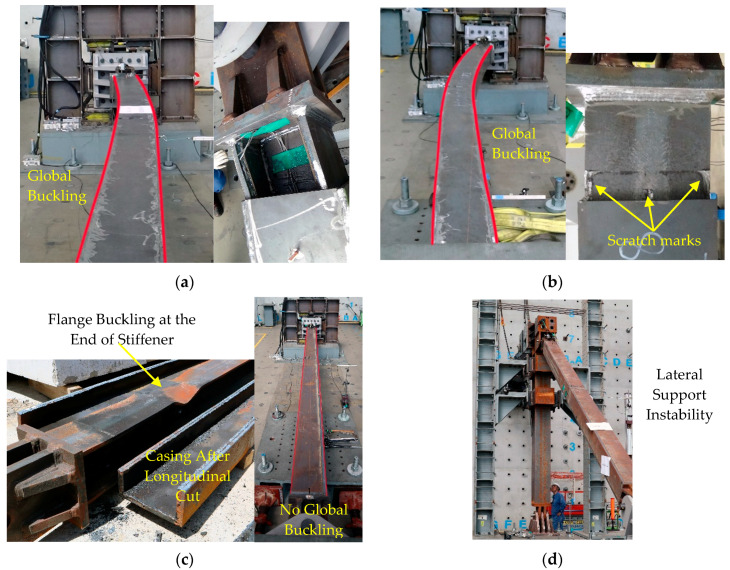
Failure mode of test specimens: (**a**) Specimen 1—global buckling and unconstrained-length deformation; (**b**) Specimen 2—global buckling and scratch marks on the unconstrained length; (**c**) Specimen 3—flange buckling and no global buckling; (**d**) Specimen 4—lateral support instability.

Figure 6 shows the load–deformation curve of the test specimens with compressive force and displacement on the positive axis. The displacements shown in Figure 6 were measured by displacement transducer 2. Figure 6a,b and Figure 7a,b demonstrate that Specimens 1 and 2 failed by global buckling, which occurred during the first compression loading of the 2.0*Δ_bm_* and 1.5*Δ_bm_* loading cycles, respectively. The maximum measured axial displacement of Specimen 1 prior to global buckling was 51 mm in compression and 68 mm in tension. Meanwhile, for Specimen 2, it was 48 mm in compression and 44 mm in Tension. Following the global buckling of Specimens 1 and 2, the sideways bending of the BRB resulted in close contact between the core and the casing; this is shown in the unconstrained length’s deformed shape and scratch marks displayed in Figure 7a,b.

Specimen 3 exhibited a stable hysteresis to the end of the first 2.0*Δ_bm_* cycle. However, during the second compression loading of the 2.0*Δ_bm_* cycle, a sudden increase in stiffness was observed, as presented in Figure 6c. The increase in stiffness was due to core flange buckling, which led to interlocking between the core flange and inner wall of the casing. The buckled shape of the core flange after the casing was removed by flame cutting and is shown in Figure 7c. The increased stiffness resulted in a force demand beyond the actuator capacity, leading to the termination of the test. 

Specimen 4, tested in the frame assembly test setup, showed stable hysteresis until the first compression loading of the 1.0*Δ_bm_* cycle. However, the test was terminated owing to lateral support frame instability, as shown in Figure 6d.

**Figure 8:**
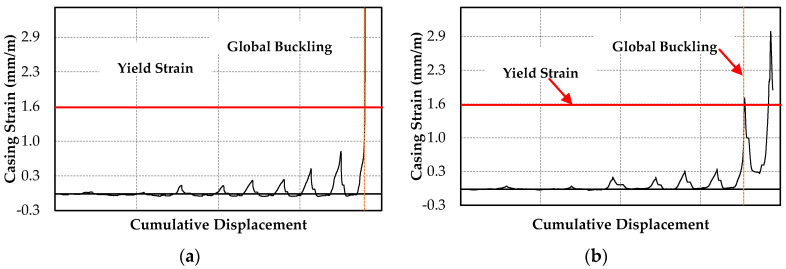

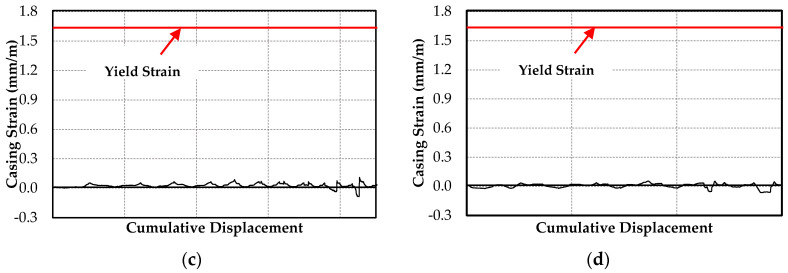
Longitudinal casing strain. Specimens (**a**) 1, (**b**) 2, (**c**) 3, and (**d**) 4.

Table 3 lists the maximum measured force (*P_max_*), displacement (*Δ_max_*), and core strain (*ε_max_*), along with the mode of failure, *P_max_/P_cr_,* and *P_max_/P_cy_*. Specimens 1 and 2 buckled under a maximum force lower than the critical buckling load, with *P_max_/P_cr_* ratios of 0.77 and 0.74, respectively. However, the maximum measured compressive forces were at the casing yielding force with *P_max_/P_cy_* ratios of 0.98 and 0.95 for Specimens 1 and 2, respectively. Figure 8a,b shows the variation of the casing longitudinal strain measured at the center of the BRB. The casing strain in Specimens 1 and 2 was below the yield strain until the onset of global buckling; this indicates that casing yielding was the cause of global buckling. This phenomenon is more noticeable in Specimen 2, in which global buckling occurred near the end of a loading cycle.

In contrast, Specimens 3 and 4 with higher *P_cr_* and *P_cy_* values did not exhibit global buckling or casing strain above the yield strain. Figure 8c and d show the casing strain variation in Specimens 3 and 4, respectively. The *P_max_/P_cr_* and *P_max_/P_cy_* ratios of Specimens 3 and 4 are presented in Table 3.

The yield strain presented in Figure 8a–d was calculated as the ratio of the casing yield strength to the modulus of elasticity (200 GPa).

## 4. Finite Element Analysis

### 4.1. Finite Element Model of Test Specimens

To better understand the NOVEL brace, an analytical study using the finite element computer program ABAQUS/CAE 2017 (Dassault Systemes Simulia Corp., Johnston, RI, USA) [18] was conducted for 14 NOVEL brace models in the subassembly setup. Previously, Specimens 1–4, called Models 1–4, respectively, were analyzed under cyclic load to validate the finite element model. In particular, elastic modulus of 200 GPa and the stress–strain relation obtained from the coupon test were converted to the true stress–strain and applied to the corresponding components of the brace. Material nonlinearity with the von Mises yield criterion was considered, and the cyclic hardening behavior was modelled using the combined isotropic and kinematic hardening model following the true stress–strain data [18,19]. All brace components were modeled with 20 node solid elements, C3D20R, and the interaction between the core and the casing was modeled as a hard contact with no penetration. Separation after contact was allowed to permit separation during tension loading. The tangential behavior of the contact was modeled through a penalty friction formulation with a friction coefficient of 0.8 to simulate the dry steel-to-steel interface.

**Figure 9:**
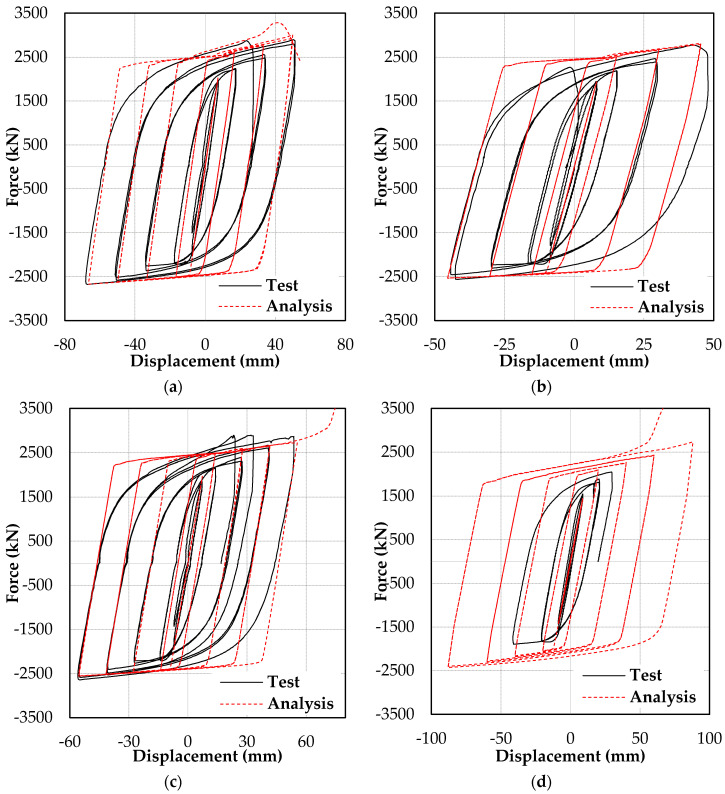
Comparison of test and finite element analysis results. Specimens (**a**) 1, (**b**) 2, (**c**) 3, and (**d**) 4.

**Figure 10:**
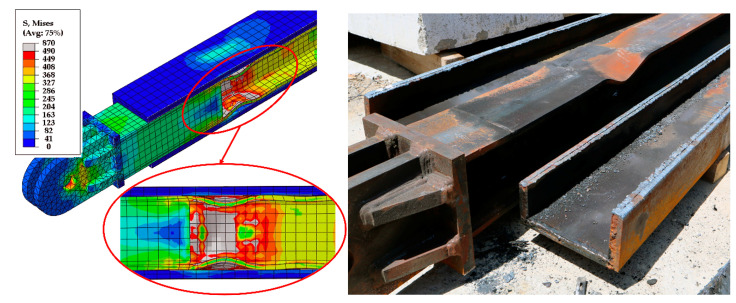
Core flange buckling of Specimen 3 in test and finite element.

The first buckling mode shape with a 12 mm magnitude (1/500 of brace length) [16] was applied as the initial imperfection in all finite element models. Figure 9 compares the hysteresis loops from the test and the finite element analysis. The finite element analysis predicted the maximum forces, initial stiffness, and post-yield stiffness of the test specimens. Moreover, the sudden stiffness increase observed in Specimen 3 and associated with core flange buckling was reflected in the finite element analysis, as shown in Figure 9c and Figure 10. Figure 10 compares the core flange buckling in the test and the finite element analysis.

**Figure 11:**
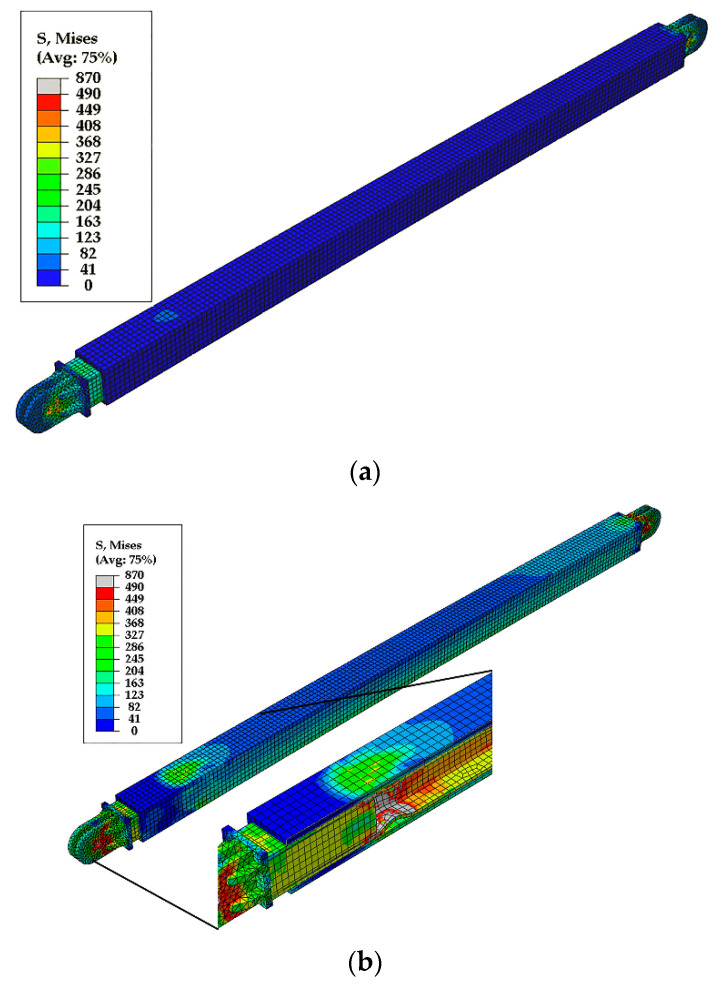
Casing stress (**a**) before and (**b**) after core flange buckling.

Figure 11 compares the casing stress before and after core flange buckling. The figure shows the increase in casing stress following flange buckling. The highest casing stress occurred in the vicinity of the buckled core flange. This indicates that the sudden increase in strength and stiffness was due to friction resistance caused by the increased contact pressure between the buckled flange and casing. The wave shape of the buckled flange increased the contact pressure, resulting in axial force transfer to the casing.

### 4.2. Parametric Study

**Figure 12:**
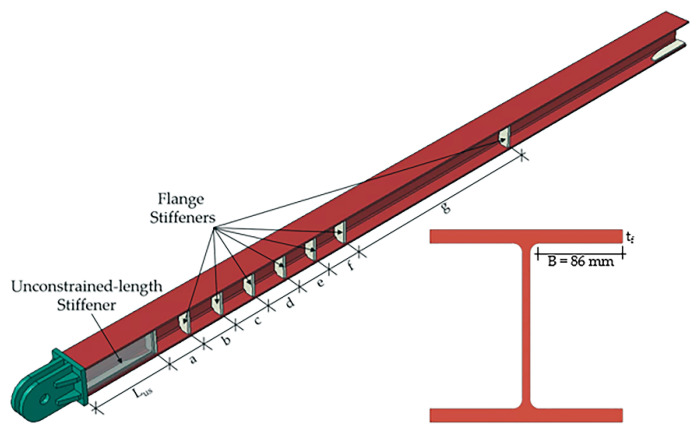
Variables of the parametric study.

A finite element parametric study was conducted to investigate the influence of stiffeners and core flange thickness on flange local buckling. Figure 12 shows the variables of the parametric study, specifically length of the unconstrained-length stiffener (*L_us_*), number and spacing of flange stiffeners distributed along the length of the core, and core flange thickness (*t_f_*). The 14 finite element models included in the parametric study are listed in Table 4. Here, L, FS, and TF in the model names indicate the study variables in that model and represent the length of the unconstrained-length stiffener, flange stiffeners, and core flange thickness, respectively. The numbers after L and TF indicate the values of *L_us_* and *t_f_* for the models. The first and second numbers after FS indicate the number of flange stiffener plates used and the total core length stiffened by flange stiffeners, respectively. The flange stiffener spacings are listed in Table 4.

**Figure 13:**
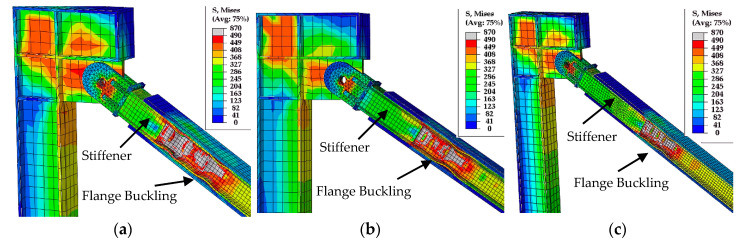

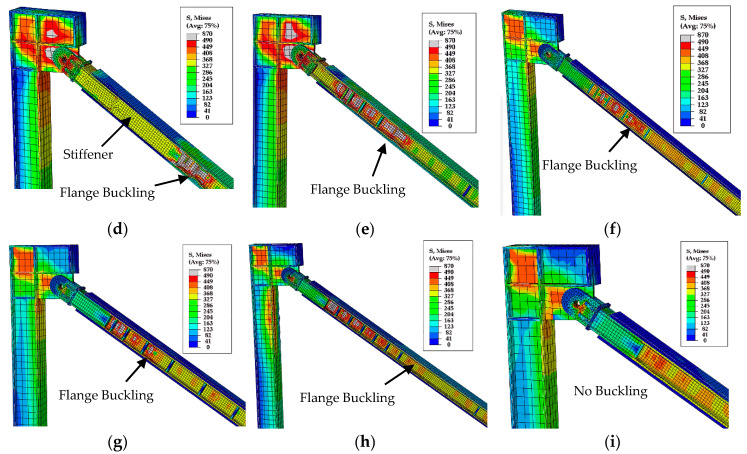
Influence of stiffener details and core flange thickness: (**a**) L340, (**b**) L510, (**c**) L850, (**d**) L1500, (**e**) FS5-2000, (**f**) FS6-2000, (**g**) FS7-2000, (**h**) FS8-3000, and (**i**) TF17.

Figure 13 shows the deformed shape of the parametric study models at the 2.0*Δ_bm_* cycle (d = 80 mm or 2.0% drift). Figure 13a–d show the influence of *L_us_*. As observed in the figures, flange buckling occurred at the end of the stiffener regardless of the stiffener length.

Figure 13e–h show the influence of flange stiffeners and stiffener spacing. As can be observed from the stress contours, the use of flange stiffeners resulted in stress concentration in the core web. Moreover, flange buckling was observed in all FS models and occurred where the stiffener spacing was 400 mm or above 400 mm in all the FS models.

**Figure 14:**
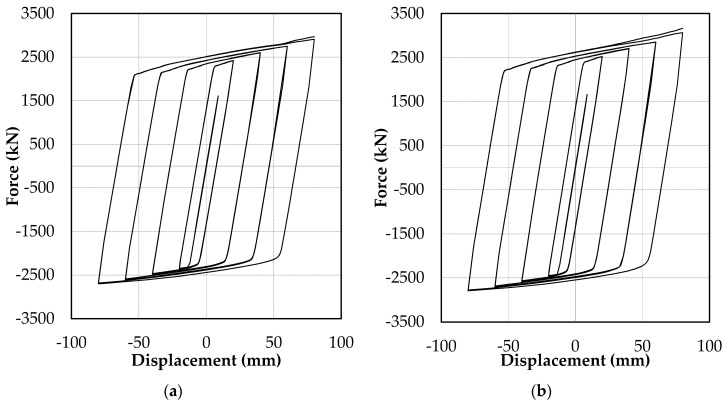
Hysteresis responses: (**a**) TF17 and (**b**) TF18.

Figure 13i shows the stress contour and deformed shape of the TF17 model. TF17 and TF18 showed no flange buckling and exhibited stable hysteresis until the end of the loading protocol. The resulting hysteresis is presented in Figure 14. The TF15 and TF16 models failed by flange buckling, similar to the L510 model. The minimum core flange slenderness ratio (B/*t_f_*) that did not result in flange buckling up to the end of the 2.0*Δ_bm_* (core strain of 1.46%) was 5.06.

## 5. Conclusions

In this study, a buckling-restrained brace named NOVEL brace was developed by utilizing a square casing and an H-section core with unconstrained length only on one side of the brace. Among the four specimens tested, two specimens with *P_cr_/P_y_* = 1.68 and *P_cy_/P_y_* = 1.31 exhibited global buckling at core strain ranging from 1.1% to 1.2% when the casing moment to casing yield moment ratio was in the range of 0.95–0.98. The test specimen with *P_cr_/P_y_* = 4.91 and *P_cy_/P_y_* = 3.74 exhibited stable hysteresis up to a core strain of 1.2% and failed by core flange buckling. Finite element analysis was conducted and validated, and a parametric study was performed to examine the effect of the unconstrained-length stiffener, flange stiffeners, spacing of flange stiffeners, and width-to-thickness ratio of the core flange. Based on the test results and the finite element parametric study, the following conclusions were drawn:

1. In test specimens with *P_cr_/P_y_* = 1.68, global buckling occurred at a compressive force equal to 74–77% of the critical buckling load when the casing moment reached 95–98% of the casing yield moment. This result indicates that global buckling occurred owing to flexure yielding of the casing, and both the stiffness and strength of the casing should be considered when proportioning a BRB.
2. In test specimens with a core flange width-to-thickness ratio of 14.28, flange buckling occurred at a core strain of 1.2%. The flange buckling resulted in friction locking between the core and the casing due to increased contact pressure in the vicinity of the buckled flange. This phenomenon led to axial force transfer to the casing and a sudden increase in stiffness.
3. The finite element parametric study indicated that increasing the flange thickness is more feasible than providing flange stiffeners or adjusting the length of the unconstrained-length stiffener. The minimum core flange width-to-thickness ratio that resulted in a stable hysteresis up to the 2.0*Δ_bm_* cycle (1.46% core strain) was 11.76.
4. Further research is needed to investigate the influence of utilizing single unconstrained length as opposed to two unconstrained lengths.

## Figures and Tables

**Table 1:** Material properties.

Member	Coupon Location	Thickness (mm)	Yield Strength, *f_y_* (MPa)	Tensile Strength, f_u_ (MPa)	Elongation (%)
Core	Flange	14	321	497	37.6
	Web	8	328	488	37.9
Casing	Wall	10	324	492	36.2

**Table 2:** Specimen strength.

Specimen No.	Core		Casing				
	*P_y_* (kN)	*P_u_* (kN)	*P_cr_* (kN)	*P_cr_/P_y_*	*M_y_* (kN·m)	*P_cy_*^1^ (kN)	*P_cy_/P_y_*
1	2249	3455	3774	1.68	189	2949	1.31
2	2249	3455	3774	1.68	189	2949	1.31
3	2249	3455	11,052	4.91	493	8412	3.74
4	2249	3455	11,052	4.91	493	8412	3.74

^1^ Brace axial force that results in flexure yielding of the casing.

**Table 3:** Test results.

Specimen No.	*P_max_* (kN)		*Δ_max_* (mm)		*ε_max_* (%)		PmaxPcr 3	PmaxPcy 4	Failure Mode
	(+) ^1^	(−) ^2^	(+)	(−)	(+)	(−)			
1	2895	2678	51.2	68.1	1.2	1.5	0.77	0.98	Global buckling
2	2774	2564	47.9	44.3	1.1	1.0	0.74	0.95	Global buckling
3	2893 ^5^	2644	53.8	55.9	1.2	1.2	0.26	0.34	Core flange buckling
4	2049	1894	29.9 ^6^	41.5	0.5	0.7	0.22 ^7^	0.29	-

^1^ Compression. ^2^ Tension. ^3^ Maximum compressive force to Euler buckling force ratio. ^4^ Maximum compressive force to casing yield force ratio. ^5^ Limited by actuator capacity. ^6^ Subassembly frame drift. ^7^ Maximum brace axial force.

**Table 4:** Details of the finite element model.

Model	*L_us_*	a	b	c	d	e	f	g	*t_f_* ^1^
L340	340	-							14
L510	510								14
L850	850								14
L1500	1500								14
FS4-3000	510	500	1000	1500	-	-	-	-	14
FS5-2000	510	100	150	200	250	-	-	-	14
FS6-2000	510	100	200	300	400	1000	-	-	14
FS7-2000	510	100	200	300	400	500	500	-	14
FS7-3000	510	250	250	250	250	500	1500	-	14
FS8-3000	510	250	250	250	250	250	250	1500	14
TF15	510	-							15
TF16	510								16
TF17	510								17
TF18	510								18

^1^ Core flange thickness.
